# *Angelica Sinensis* Polysaccharides Stimulated UDP-Sugar Synthase Genes through Promoting Gene Expression of *IGF-1* and *IGF1R* in Chondrocytes: Promoting Anti-Osteoarthritic Activity

**DOI:** 10.1371/journal.pone.0107024

**Published:** 2014-09-09

**Authors:** Yinxian Wen, Jing Li, Yang Tan, Jun Qin, Xianfei Xie, Linlong Wang, Qibing Mei, Hui Wang, Jacques Magdalou, Liaobin Chen

**Affiliations:** 1 Department of Orthopedic Surgery, Zhongnan Hospital of Wuhan University, Wuhan, China; 2 Department of pharmacology, Basic Medical School of Wuhan University, Wuhan, China; 3 Department of Pharmacology, School of Pharmacy, The Fourth Military Medical University, Xi’an, China; 4 Hubei Provincial Key Laboratory of Developmentally Originated Disease, Wuhan, China; 5 UMR 7365 CNRS-Université de Lorraine, Faculté de Médecine, Vandœuvre-lès-Nancy, France; King’s College London, United Kingdom

## Abstract

**Background:**

Osteoarthritis (OA) is a chronic joints disease characterized by progressive degeneration of articular cartilage due to the loss of cartilage matrix. Previously, we found, for the first time, that an acidic glycan from *Angelica Sinensis* Polysaccharides (APSs), namely the APS-3c, could protect rat cartilage from OA due to promoting glycosaminoglycan (GAG) synthesis in chondrocytes. In the present work, we tried to further the understanding of ASP-3c’s anti-OA activity.

**Methodology/Principal Findings:**

Human primary chondrocytes were treated with APS-3c or/and recombinant human interleukin 1β (IL-1β). It turned out that APS-3c promoted synthesis of UDP-xylose and GAG, as well as the gene expression of UDP-sugar synthases (USSs), insulin like growth factor 1 (*IGF1*) and IGF1 receptor (*IGF1R*), and attenuated the degenerative phenotypes, suppressed biosynthesis of UDP-sugars and GAG, and inhibited the gene expression of *USSs*, *IGF1* and *IGF1R* induced by IL-1β. Then, we induced a rat OA model with papain, and found that APS-3c also stimulated GAG synthesis and gene expression of *USSs*, *IGF1* and *IGF1R in vivo*. Additionally, recombinant human IGF1 and IGF1R inhibitor NP-AEW541 were applied to figure out the correlation between stimulated gene expression of USSs, *IGF1* and *IGF1R* induced by APS-3c. It tuned out that the promoted GAG synthesis and USSs gene expression induced by APS-3c was mediated by the stimulated *IGF1* and *IGF1R* gene expression, but not through directly activation of IGF1R signaling pathway.

**Conclusions/Significances:**

We demonstrated for the first time that APS-3c presented anti-OA activity through stimulating *IGF-1* and *IGF1R* gene expression, but not directly activating the IGF1R signaling pathway, which consequently promoted UDP-sugars and GAG synthesis due to up-regulating gene expression of USSs. Our findings presented a better understanding of APS-3c’s anti-OA activity and suggested that APS-3c could potentially be a novel therapeutic agent for OA.

## Introduction

Osteoarthritis (OA) is a chronic joint disease with a high prevalence in elderly people, which is characterized by progressive degradation of articular cartilage [1], [2]. An imbalance between anabolism and catabolism of chondrocytes leads to loss of matrix components including collagens and glycosaminoglycan (GAG) chains, which consequently triggers the continuous degradation of articular cartilage [3]. Current pharmacological therapies for OA are mainly analgesics, nonsteroidal anti-inflammatory drugs and other symptomatic slow-acting drugs (SYSADOA), which mainly act on pain relief and mobility improvement but with dissatisfactory effects on alleviating cartilage degeneration [4], [5], [6].

*Angelica sinensis* is a traditional herbal medicine that has long been applied to relieve the pain and slow the progress of OA. But what the therapeutic ingredient is and how it works still remains unclear. *Angelica sinensis* polysaccharides (APSs) are natural polysaccharides extracted from the root of *Angelica sinensis*, which have been proved beneficial in multiple disease models including cancer, ischemia-reperfusion, inflammation, leukopenia, ulcerative colitis and hepatic injury [7], [8], [9], [10], [11], [12], [13]. APS-3c is an acidic glycan from APSs [14]. In the previous work, we found, for the first time, that ASP-3c could protect rat cartilage from OA due to promoting GAG synthesis [15]. However, more detailed study on the anti-OA activity of APS-3c is still needed.

In mammals, GAG synthesis starts with the step-wise addition of carbohydrate moieties from the corresponding high energy donors, uridine diphosphate sugars (UDP-sugar) onto the serine residues of the core protein by certain glycosyltransferases (GTs) [16]. These compounds are the products of a series of UDP-sugar synthases (USSs). The UDP-xylose synthase 1 (UXS1) catalyzes the formation of UDP-xylose (UDP-xyl) through decarboxylation of UDP-glucuronic acid (UDP-GlcA) [17], the product of UDP-glucose 6-dehydrogenase (UGDH) from UDP-glucose [18]. In the Leloir pathway of galactose metabolism, galactose-1-phosphate uridylyltransferase (GALT) catalyzes the conversion of UDP-glucose and UDP-galactose [19], while UDP-galactose-4-epimerase (GALE) catalyzes the reversible conversion of UDP-galactose to UDP-glucose and from Uridine diphospho-N-acetylglucosamine (UDP-GlcNAc) to UDP-N-acetylgalactosamine (UDP-GalNAc) [19]. All these UDP-sugars are essential for GAG chain elongation and/or sorting [20].

The process of GAG synthesis in articular cartilage is regulated by multiple cytokines and growth factors, among which are interleukin β (IL-1β) and insulin like growth factor 1 (IGF-1) [21], [22]. As a typical pro-inflammatory factor in OA pathophysiology, IL-1β could inhibit synthesis of GAG due to decrease gene expression and enzyme activity of GTs [23], [24]. On the contrary, IGF-1 is one of the key protective factors of cartilage. It reported that IGF-1 could attenuate the catabolic and degenerative changes induced by IL-1β in chondrocytes [25]. The binding of IGF1 to IGF1 receptor (IGF1R) leads to the autophosphorylation of the receptor, and subsequently induces insulin receptor substrates (IRS) and Src homology/collagen domain protein, which consequently activate the PI3K/Akt and MEK/ERK signaling pathways [26], [27], [28]. However, IGF-1 enhances GAG synthesis in human primary chondrocyte mainly through the PI3K/Akt pathway but not the MEK/ERK pathway [29].

To further investigate the possible mechanism involved in the anti-OA activities of APS-3c, we tested the effects of APS-3c on UDP-sugar synthesis *in vitro*, as well as the gene expression of USSs, *IGF-1* and *IGF1R* both *in vivo* and *in vitro*. Then, exogenous IGF-1, IL-1β and IGF1R inhibitor NVP-AEW541 were used to figure out whether IGF-1 and IGF1R mediated the promotion of APS-3c on the USSs gene expression and the consequent GAG synthesis in human primary chondrocytes. As such, this study would contribute to a better understanding of the anti-OA activities of APS-3c.

## Materials and Methods

### Materials

DMEM/F12 (1∶1) and fetal bovine serum (FBS) were supplied by Thermo Scientific (Beijing, China). Recombinant human IL-1β and IGF-1 were bought from PeproTech (NJ, USA). Cell Counting Kit-8 (CCK-8) was acquired from Dojindo (Kumamoto, Japan). Collagenase type II, Chondroitin sulfate sodium salt from shark cartilage, 1,9-dimethylmethylene Blue (DMB), Alcian blue dye and Uridine 5′-diphospho-N-acetylglucosamine sodium salt (UDP-GlcNAc) were obtained from Sigma-Aldrich (MO, USA). UDP-xyl was obtained from CarboSource Services (GA, USA). Ultrafiltration membranes were obtained from Millipore (MA, USA). CarboPac PA20 Carbohydrate Column was supplied by Dionex (CA, USA). TRIzol reagent was obtained from Life Technologies (NY, USA). First Strand cDNA Synthesis Kit and real time quantitative polymerase chain reaction (RT-PCR) kits were purchased from Takara Biotechnology (Dalian, China). Oligonucleotide primers were synthesized by Sangon Biotech (Shanghai, China). Anti-GALE, anti-GALT, anti-UGDH, anti-UXS1, anti-IGF1 and anti-GAPDH polyclonal antibodies were obtained from Proteintech (CHI, USA). Anti-IGF1R and anti-IRS1 polyclonal antibodies were obtained from Santa Cruz Biotechnology (CA, USA). Anti-phosphorylated-IRS1 (Y612) antibody was supplied by Abcam (Cambridge, UK). Western Bright ECL HRP substrate was purchased from Advansta (CA, USA). RIPA lysis buffer and BCA Protein Assay Kit were purchased from Beyotime Institute of Biotechnology (Haimen, China). NVP-AEW541 was obtained from Novartis Pharma AG (Basel, Switzerland). APS-3c was a gift from Professor Mei Qibing from the Fourth Military Medical University of China [14]. Other chemicals and reagents were of analytical grade.

### Cartilage specimen

Human articular cartilage was obtained in total knee replacement surgery from patients (11 knees of 8 female patients, aged 66±10 years), who were diagnosed with OA using the criteria of the American College of Rheumatology for OA [30]. The protocol was in accordance to the ethical guidelines of the Declaration of Helsinki and approved by Medical Ethics Committee of the Zhongnan Hospital of Wuhan University (Approval Number, 2012030). Informed consent was obtained from each donor as written form.

Pathogen-free male adult Wistar rats (weighed 220–280 g) were supplied by Experimental Centre of Medical Scientific Academy of Hubei province, which also approved animal study protocol applied in the study (No. 2008–0005). The protocol was in accordance with the Guide for the Care and Use of Laboratory Animals (eighth edition) by the National Research Council of the United States National Academies. The animal study was performed in the Animal Biosafety Level 3 Laboratory of Wuhan University (Wuhan, China) accredited by the AAALAC International. The OA model was induced as described previously [15]. Then, all the rats were anaesthetized using isoflurane through inhalation, and subsequently sacrificed by cervical dislocation for the knee joints.

### Histopathology assay

Cartilage samples from the weight-bearing area of the knee joint were applied in pathological test, which were fixed in 4% paraformaldehyde overnight and embedded in paraffin wax, successively. Then, cartilage sections of 5 µm were obtained perpendicularly to the surface of articular cartilage. Safranin O staining was performed according to the standard protocol. Moreover, protein level of UGDH, GALE, IGF-1 and IGF1R of the cartilage was also detected using immunohistochemistry (IHC) assay as previously described [31], [32]. And relative protein level was presented as mean optical density (MOD) of each chondrocytes using NIS-elements software (Nikon, Tokyo, Japan).

### Isolation and treatment of human primary chondrocytes

Chondrocytes were obtained from macroscopically normal areas of cartilage and cultured as a monolayer using DMEM/F12 (1∶1) with 10% (v/v) fetal bovine serum, 100 IU/ml penicillin, 100 µg/ml streptomycin, and 2 mM glutamine at 37°C with 5% CO_2_. Then, chondrocytes were treated with 2, 10 and 50 µg/ml APS-3c (or 2, 10 and 50 ng/ml IGF-1) alone for 48 h, or pre-treated with 10 ng/ml IL-1β for 30 min and subsequently co-treated with the IL-1β and 2, 10 and 50 µg/ml APS-3c (or 50 ng/ml IGF1) for another 48 h, to detect the GAG synthesis and USSs gene expression. Chondrocytes were also treated with 50 ng/ml IGF1 or 10 µg/ml APS-3c for 30 min, or pre-treated with 1 µM NVP-AEW541 for 30 min and then co-treated with the NVP-AEW541 and 10 µg/ml APS-3c or NVP-AEW541 and 50 ng/ml IGF1 for another 30 min, to detect the phosphorylated IRS-1. Moreover, for the assay of GAG synthesis and USSs gene expression, chondrocytes were also pre-treated with 1 µM NVP-AEW541 for 30 min and then co-treated with the NVP-AEW541 and 10 µg/ml APS-3c for another 48 h.

### Cell viability assay

Chondrocytes were cultured in 96-well plates at 2×10^4^ cells/ml and treated as described above. Then, the medium was replaced with 100 µl serum-free DMEM/F12 containing 10 µl CCK-8 reagent. Absorbance of each well was determined at 450 nm using a UV-1601 spectrophotometer (Shimadzu, Kyoto, Japan).

### Transmission electron microscopy

Transmission electron microscopy observation was performed as previously described [33]. The chondrocytes were fixed in Karnovsky’s fixative for 30 min and post-fixed in a 1% OsO4 solution. Then, samples were dehydrated in increasing ethyl alcohol concentrations, embedded in Epon, and cut on a LKB-V ultramicrotome (Bromma, Kista, Sweden). 2% (w/v) uranyl acetate/lead citrate was used for contrasting these ultrathin sections. Then, the ultrastructures of chondrocytes were observed and photographed using a H-600 TEM (Hitachi, Tokyo, Japan) at a magnification of 15,000.

### GAG assay

Both supernatant and chondrocyte-associated GAG were collected using papain extraction reagent and dyed using DMB color reagent as reported [34], [35]. Absorbance was detected using a UV-1601 spectrophotometer. A standard curve constructed using chondroitin sulfate sodium salt was applied to quantify the total GAG of the chondrocyte cultures. Total protein of the cultures was also quantified to calibrate the total GAG of each chondrocyte culture. Then, Chondrocytes cultured on coverslips were fixed in 10% (w/v) neutral formalin for 15 min at room temperature, stained overnight at 4°C with 0.5% (w/v) Alcian blue dye and photographed using an AZ100 Microscopes (Nikon, Tokyo, Japan). MOD of each chondrocyte was obtained using NIS-Elements software.

### Real time quantitative PCR assay

Total RNA was isolated by TRIzol reagent following the manufacturer’s protocol. Single-strand cDNA was obtained from purified total RNA using the reverse transcription kit. Primers used in this study were designed using Primer Premier 5.0 (Premier Biosoft, CA, USA) and the NCBI BLAST database. Primer sequences and the optimal PCR conditions were shown in Table 1. RT-PCR assay was performed on a StepOne thermal cycler (Applied Biosystems, NY, USA) following the procedure: pre-denaturation at 95°C for 30 sec, denaturation at 95°C for 5 sec, annealing at Tm for 30 sec, and extension at 72°C for 30 sec. The last 3 steps ran for 40 cycles. Relative standard curves were applied to quantify the mRNA level of each sample, while GAPDH mRNA level was detected and used as internal reference.

**Table 1:** Primer sequences and the optimal PCR conditions.

Genes	Accession number	Sequence	Product size (bp)	Tm (°C)
*GALE*	NM_000403	F: 5′-GGCAGACAAGACTTGGAACGC-3′	131	58
		R: 5′-TCGCCACCTGGGAGACATAA-3′		
*GALT*	NM_000155.3	F: 5′-AGCGTGATGATCTAGCCTCCA-3′	217	60
		R: 5′-GCAAGCATTTCGTAGCCAACC-3′		
*UGDH*	NM_003359.3	F: 5′-CAGGCTATGTTGGAGGACCC-3′	162	60
		R: 5′-TCGACAGGATTCTACCACTTCTT-3′		
*UXS1*	NM_001253875.1	F: 5′-TCCCGCTGGAGGAAGGTTTA-3′	101	60
		R: 5′-TCTGGCAGGCTTTGGTTTGG-3′		
*IGF-1*	NM_001111285.1	F: 5′-GATGTATTGCGCACCCCTCA-3′	168	60
		R: 5′-TTCTGTTCCCCTCCTGGATGT-3′		
*IGF1R*	NM_000875.3	F: 5′-GAGAACATGGAGAGCGTCCC-3′	220	60
		R: 5′-CCAAGGATCAGCAGGTCGAA-3′		
*GAPDH*	NM_002046.4	F: 5′-GAAATCCCATCACCATCTTCCAG-3′	313	60
		R: 5′-GAGTCCTTCCACGATACCAAAG-3′		

RT-PCR, real time quantitative polymerase chain reaction; *GALE*, UDP-galactose-4-epimerase; *GALT*, galactose-1-phosphate uridylyltransferase; *UGDH*, UDP-glucose 6-dehydrogenase; *UXS1*, UDP-xyl synthase 1; *IGF-1,* insulin like growth factor 1; *IGF1R*, IGF-1 receptor; *GAPD*, glyceraldehyde-3-phosphate dehydrogenase.

### Western blotting analysis

Total proteins were obtained using RIPA lysis buffer and quantified using a BCA kit following the protocol. Then, total proteins were loaded as 40 µg per lane, separated by SDS-PAGE (5 and 10% gels) and blotted onto nitrocellulose membranes. Membranes were blocked in 5% skimmed milk, probed with anti-human GALE (1∶1000), GALT (1∶500), UGDH (1∶1000), UXS1 (1∶800), IGF-1 (1∶800), IGF1R (1∶1000), IRS1(1∶1000), phosphorylated IRS1 (Y612, 1∶500) and GAPDH (1∶1000) primary antibodies, incubated with horseradish peroxidase-conjugated secondary antibody (goat anti-rabbit IgG, 1∶5000) and visualized using ECL HRP substrate. Then, relative protein level was standardized with normal control and the GAPDH protein level obtained using Quantity One software (Version 4.6, Bio-Rad Laboratories Inc., CA, USA).

### UDP-sugars detection

High-performance anion-exchange chromatography (HPEAC) assay was performed to detect the UDP-sugars content in chondrocytes. UDP-sugar samples were obtained as previously described [36], [37]. Protein concentration of each sample was detected and adjusted to 100 µg/µl. UDP-xyl and UDP-GlcNAc were used as standards. Then, contents of UDP-xyl and UDP-GlcNAc in each chondrocyte culture were detected using a CarboPac PA20 carbohydrate column with a loading quantity of 20 µl. The gradient elution assay was performed as reported [36]. The UDP-sugar contents of each sample were presented as peak areas.

### Statistical analysis

Data analysis was performed using SPSS 17.0 (SPSS Science Inc., CHI, USA) and Prism 5.0 (GraphPad Software, CA, USA). Results were presented as mean ± S.E.M. Analysis of variance (ANOVA) and Student *t* test were applied in the study. Statistical significance was defined as *P*<0.05.

## Results

### Promoted GAG and UDP-sugars synthesis of human primary chondrocyte by APS-3c

**Figure 1:**
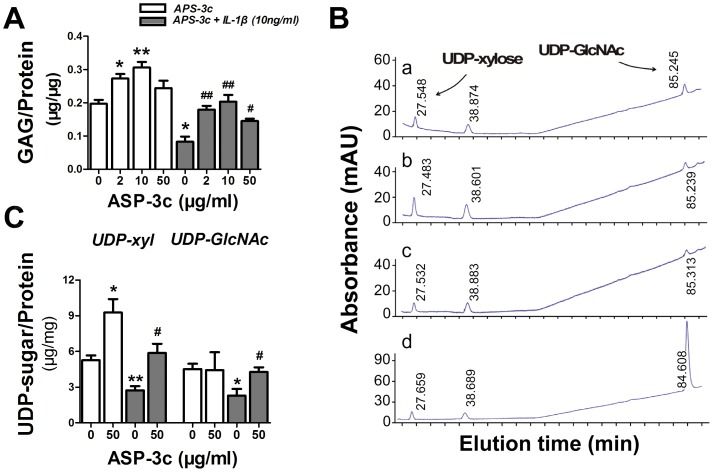
APS-3c stimulated glycosaminoglycan (GAG) and UDP-sugar synthesis *in vitro*. A, Total GAG were detected in chondrocytes treated with 2,10 and 50 µg/ml ASP-3c, or pre-treated with 10 ng/ml interleukin 1 beta (IL-1β) for 30 min and consequently co-treated with IL-1β and ASP-3c for another 48 h using DMB assay. B, UDP-sugar content in chondrocytes was also detected with high-performance anion-exchange chromatography using a CarboPac PA20 carbohydrate column. a, control; b, chondrocytes treated with 50 µg/ml ASP-3c for 48 h; c, chondrocytes treated with 10 ng/ml IL-1β for 48 h; d, chondrocytes pre-treated with 10 ng/ml IL-1β for 30 min, and subsequently co-treated with the IL-1β and 50 µg/ml ASP-3c for another 48 h. C, UDP-sugar contents were presented as peak areas compared with standards and normalized to the total protein. Values were presented as mean ± S.E.M. from three independent experiments. mAU, milli absorbance unit. *P<0.05 *versus* control group;^ #^ *P*<0.05, ^##^ *P*<0.01 *versus* IL-1β group.

Total GAG contents in human primary chondrocytes cultures treated with APS-3c were 38.4% (2 µg/ml) and 55.3% (10 µg/ml) higher than the control (Figure 1A, *P*<0.05), while the chondrocyte-associated GAG contents were also elevated up to 165.4% (50 µg/ml) of the control in APS-3c groups (Figure S1, *P*<0.05). Although IL-1β significantly suppressed GAG synthesis of the chondrocytes, APS-3c significantly inhibited the suppression of IL-1β on the total GAG contents by 116.7% (2 µg/ml), 145.9% (10 µg/ml) and 75.5% (50 µg/ml) after 48 h (Figure 1A, *P*<0.05), and the chondrocyte-associated GAG contents by 78.4% (2 µg/ml), 134.3% (10 µg/ml) and 196.5% (50 µg/ml) as well (Figure S1, *P*<0.05), compared with the IL-1β groups. Meanwhile, intracellular content of UDP-xyl and UDP-GlcNAc, the substrates for GAG synthesis, was detected using HPEAC assay, with the elution time around 27 min and 85 min, respectively (Figure 1B). The content of UDP-xyl but not UDP-GlcNAc inside the chondrocytes was 76.3% higher in APS-3c group than the control group. Both UDP-xyl and UDP-GlcNAc content were decreased in IL-1β group, to 51.8% and 50.6% of the corresponding control, respectively. However, subsequent APS-3c treatment significantly inhibited the IL-1β-induced decrease of UDP-xyl content by 115.4% and UDP-GlcNAc content by 87.1%, compared with the IL-1β group (Figure 1C, *P*<0.05).

### Sitimulated USSs gene expression *in vitro* and *in vivo* by APS-3c

**Figure 2:**
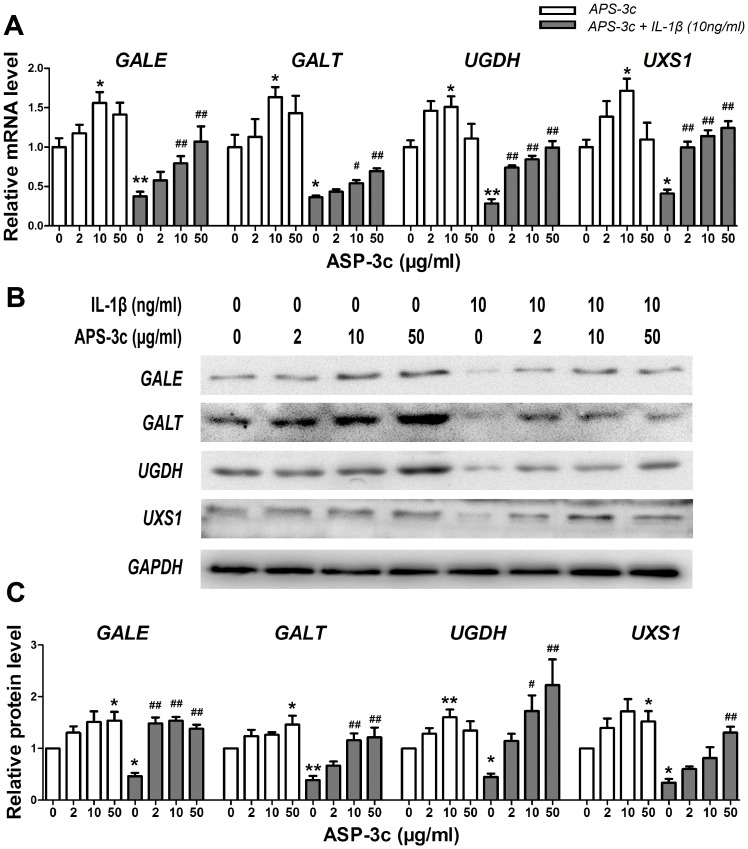
APS-3c modulated gene expression of UDP-sugar synthases *in vitro*. A, The mRNA levels of UDP-galactose-4-epimerase (*GALE*), galactose-1-phosphate uridylyltransferase (*GALT*), UDP-glucose 6-dehydrogenase (*UGDH*), and UDP-xyl synthase 1 (*UXS1*) were detected in chondrocytes treated with 2,10 and 50 µg/ml ASP-3c, or pre-treated with 10 ng/ml interleukin 1 beta (IL-1β) for 30 min and consequently co-treated with IL-1β and ASP-3c for another 48 h using real time quantitative PCR assay. B and C, The protein level of GALE, GALT, UGDH and UXS1 was also detected using Western blotting assay. Then, the mean optical density of each lane was analyzed. Values were presented as mean ± SEM from three independent experiments. *P<0.05, **P<0.01 *versus* control group;^ #^ *P*<0.05, ^##^ *P*<0.01*versus* IL-1β group.

**Figure 3:**
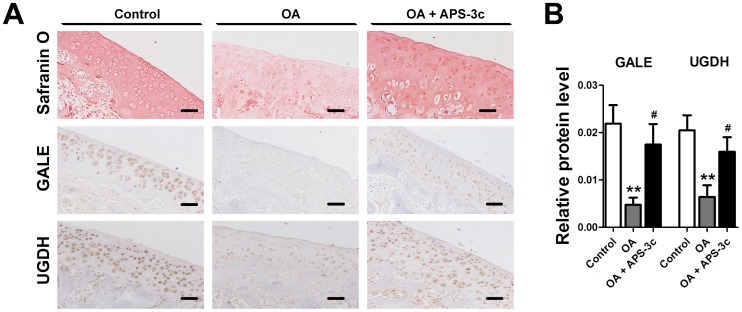
*Angelica sinensis* polysaccharides (APS-3c) increased protein expression of UDP-sugar synthases *in vivo*. A, Glycosaminoglycan content of rat cartilage from knee joints was detected using Safranin O staining. The protein expression of UDP-galactose-4-epimerase (*GALE*) and UDP-glucose 6-dehydrogenase (*UGDH*) was detected in the cartilage using immunohistochemistry. All the sections were photographed using NIS-Elements software (Nikon, Tokyo, Japan). Scale bars, 100 µm. B, Relative protein level of GALE and UGDH was presented as mean optical density of each chondrocyte. **P<0.01 *versus* control;^ #^ *P*<0.05, ^##^ *P*<0.01*versus* osteoarthritis (OA) group.

Gene expression and protein levels of *GALE*, *GALT*, *UGDH* and *UXS1* were seen to increase respectively by 56.2%, 63.5%, 50.9%, 71.6% and 54.0%, 46.0%, 60.2%, 51.2% in human primary chondrocytes after 48 h treatment with APS-3c at a concentration of 10 µg/ml or 50 µg/ml (Figure 2 *P*<0.05). While IL-1β induced a markedly suppression of those genes by more than 50%, the subsequent APS-3c treatment led to a multiplied increased of the USSs expression in a concentration-dependent manner (Figures 2, *P*<0.05). Moreover, GALE and UGDH protein expression in rat OA cartilage was markedly lower than that of normal cartilage by 78.1% and 68.6%, respectively (Figure 3, *P*<0.05), which was accompanied by the decrease of GAG content. However, obviously improvements in the degenerative features and a markedly increase in the GAG contents of the rat OA cartilage were observed. Meanwhile, protein level of GALE and UGDH were increased in APS-c group to 364.6% and 247.8% of the control group, respectively (Figure 3, *P*<0.05).

### Up-regulated IGF-1 and IGF1R gene expression *in vitro* and *in vivo* by APS-3c

**Figure 4:**
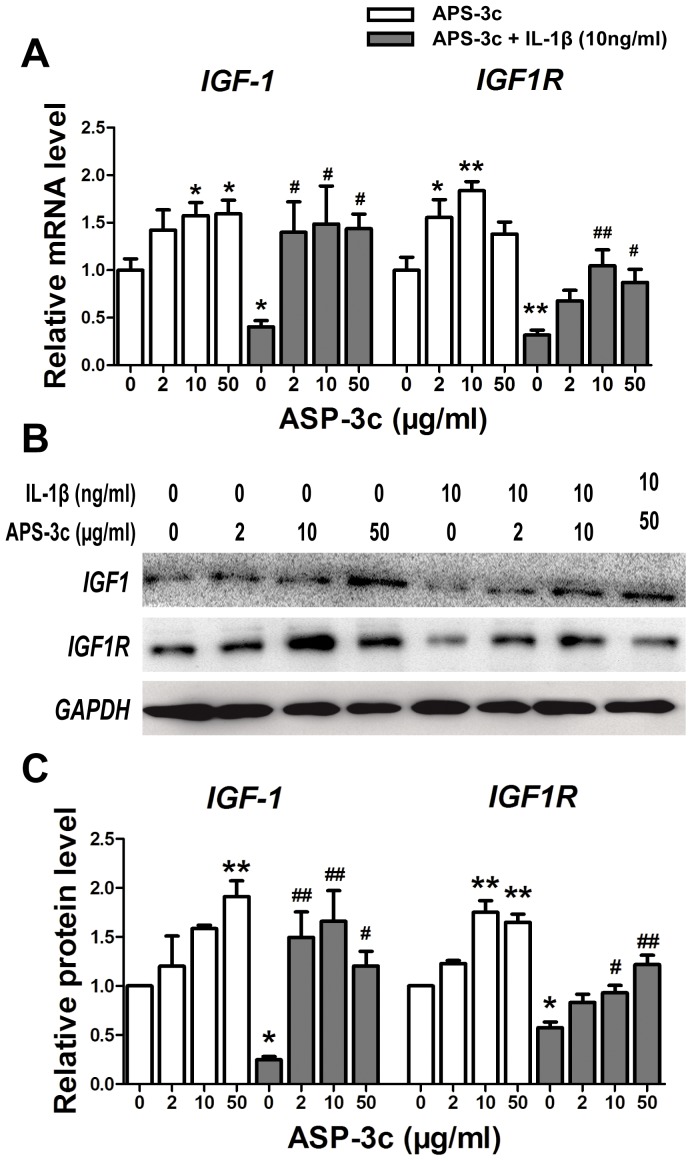
APS-3c stimulated expression of insulin like growth factor (IGF1) and its receptor (IGF1R) *in vitro*. The mRNA and protein level of IGF1 and IGF1R were detected in human primary chondrocytes treated with ASP-3c (2,10 and 50 µg/ml) or/and interleukin 1 beta (IL-1β) for 48 h. Values were presented as mean ± SEM from three independent experiments. *P<0.05, **P<0.01 *versus* control group;^ #^ *P*<0.05, ^##^ *P*<0.01*versus* IL-1β group.

**Figure 5:**
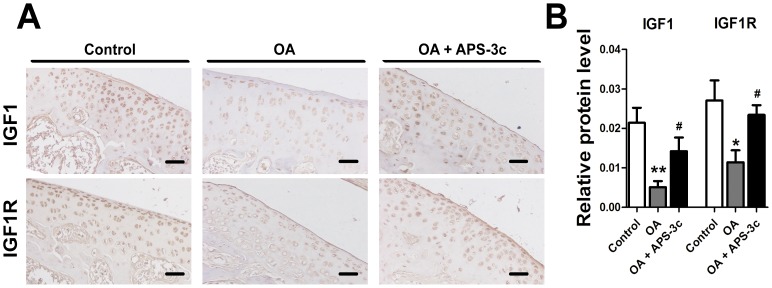
APS-3c stimulated expression of insulin like growth factor (IGF1) and its receptor (IGF1R) *in vivo*. Protein expression of IGF1 and IGF1R were detected in rat cartilage and relative protein level of IGF1 and IGF1R was presented as mean optical density of each chondrocyte. Scale bars, 100 µm. **P<0.01 *versus* control;^ #^ *P*<0.05, ^##^ *P*<0.01*versus* osteoarthritis (OA) group.

As one of the most important cartilage-protective growth factor in OA, gene expression of *IGF-1* and its receptor drew our attention. It turned out that the mRNA level of *IGF-1* and *IGF1R* was increased in APS-3c-treated chondrocytes up to 159.3% (50 µg/ml) and 183.7% (10 µg/ml), and the protein level to 192.4% (50 µg/ml) and 180.7% (10 µg/ml) of the control (Figure 4, *P*<0.05). However, both the mRNA and protein level of *IGF1* and *IGF1R* were significantly inhibited by IL-1β (Figure 4, *P*<0.05). Then, remarkable increases up to 366.7% and 328.7% in the mRNA levels, and 687.6% and 199.3% in the protein levels, of *IGF-1* and *IGF1R* were detected in chondrocytes pre-treated with IL-1β and then co-treated with IL-1β and APS-3c, when compared with the IL-1β-treated chondrocytes (Figure 4, *P*<0.05). Meanwhile, both *IGF1* and *IGF1R* protein level in rat OA cartilage was decreased to 23.8% and 42.1% of the control, which was accompanied by the decrease of GAG content and USSs protein level in the cartilage (Figure 5, *P*<0.05). However, the cartilage protein level of *IGF1* and *IGF1R* in rats from the APS-3c group was 178.4% and 105.4% higher than the OA group, respectively (Figure 5, *P*<0.05).

### Gene expression of USSs induced by exogenous recombinant human IGF-1

**Figure 6:**
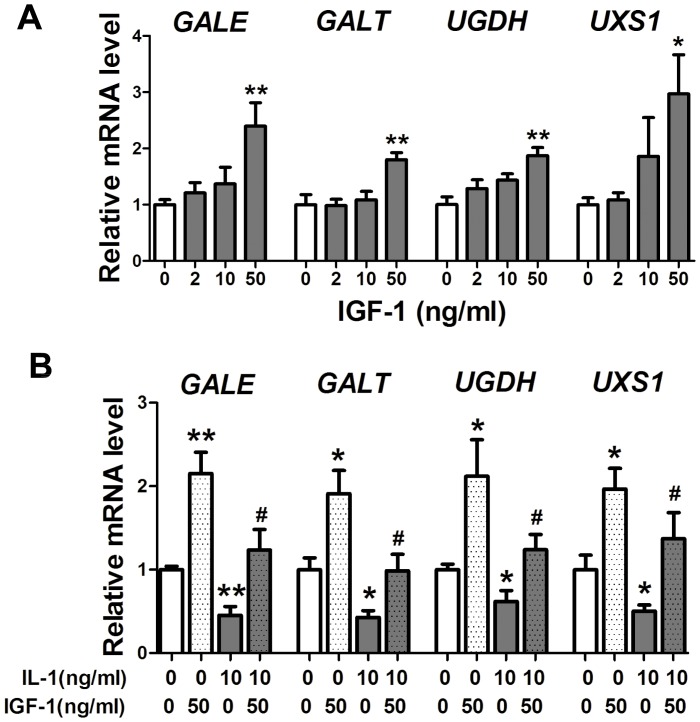
Insulin like growth factor (IGF1) modulated UDP-sugar synthases (USSs) gene expression *in vitro*. A, The mRNA expression of the USSs was detected in human primary chondrocytes treated with recombinant human IGF1 (2, 10 and 50 ng/ml) for 48 h. B, Chondrocytes were pre-treated with 10 ng/ml interleukin 1beta (IL-1β) for 30 min, then co-treated with the IL-1β and 50 ng/ml IGF1 for 48 h. Then, mRNA level of the USSs was detected. Values were presented as mean ± SEM from at least two independent experiments. *P<0.05 *versus* control group;^ #^ *P*<0.05 *versus* IL-1β group.

To uncover whether the promotion of USSs gene expression by APS-3c was related to the up-regulated IGF-1 gene expression, we also detected USSs gene expression in chondrocytes treated with exogenous IGF-1 and/or IL-1β. It turned out that mRNA expression level of *GALE*, *GALT*, *UGDH* and *UXS1* was up to 139.4%, 79.7%, 87.3% and 196.7% higher in the IGF-1 groups compared with the control, while IGF-1 also significantly up-regulated the IL-1β-inhibited USSs mRNA level to 271.8%, 230.0%, 200.4% and 271.8% of the IL-1β group (Figure 6, *P*<0.05), which was synchronous with the GAG synthesis and secretion modulated by exogenous IGF1 (Figure S2).

### NVP-AEW541 attenuated the stimulation of USSs gene expression by APS-3c

**Figure 7:**
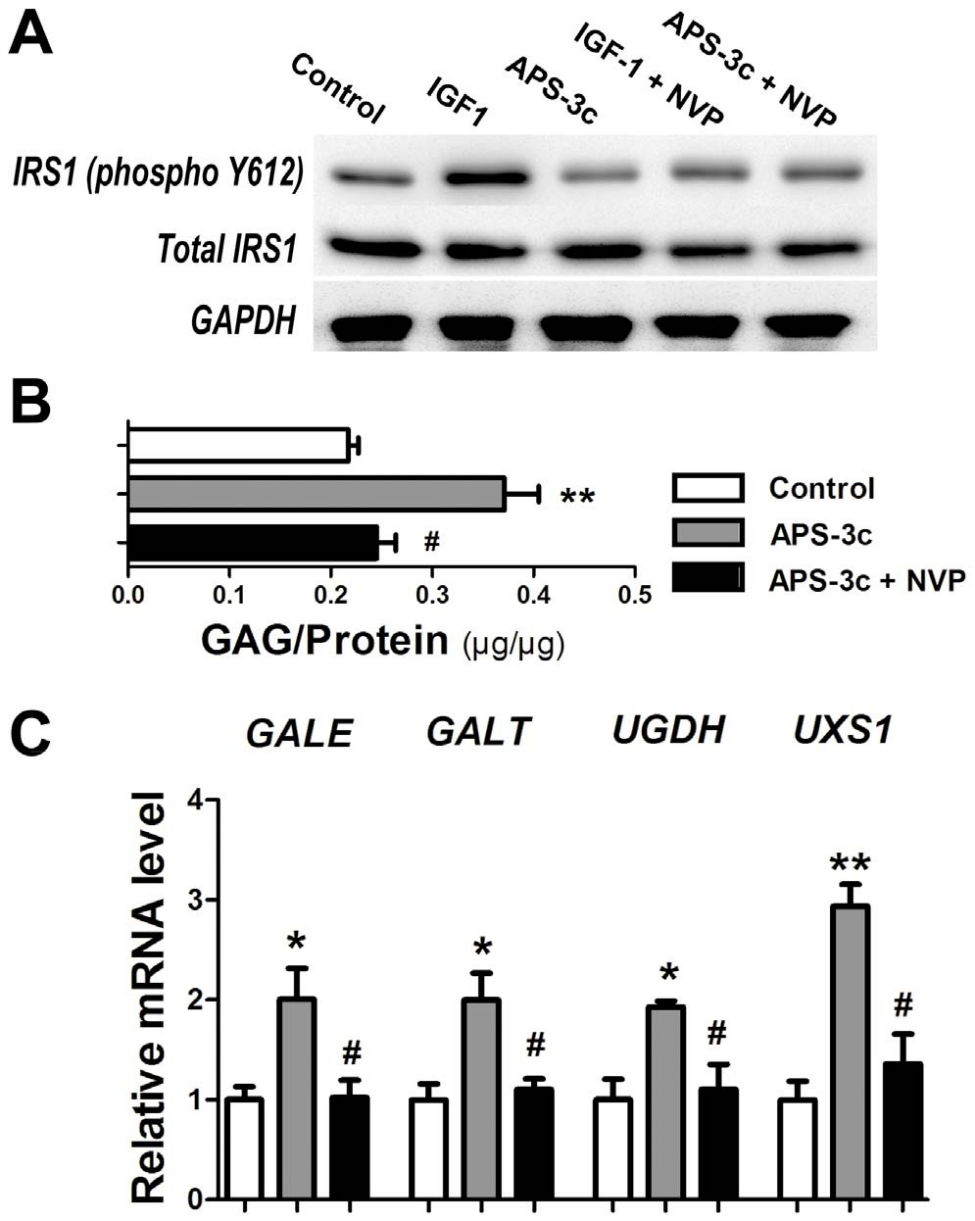
NVP-AEW541 attenuated GAG synthesis and UDP-sugar synthases (USSs) gene expression induced by APS-3c. A, Phosphorylated and total insulin receptor substrate 1 (IRS-1) was detected in the chondrocytes treated with 50 ng/ml recombinant human insulin like growth factor 1 (IGF-1) or 10 µg/ml *Angelica sinensis* polysaccharides (APS-3c) for 30 min, or pretreated with IGF1 receptor (IGF1R) inhibitor 1 µM NVP-AEW541 for 30 min and subsequently co-treated with IGF1 and NVP-AEW541 or APS-3c and NVP-AEW541 for another 30 min. B and C, Total GAG and mRNA level of the USSs was assayed in the chondrocytes treated with 10 µg/ml APS-3c for 48 h, or pretreated with 1 µM NVP-AEW541 for 30 min and subsequently co-treated with APS-3c and NVP-AEW541 for another 48 h. Values were presented as mean ± SEM from at least two independent experiments. *P<0.05 and **P<0.01 *versus* control group;^ #^ *P*<0.05 *versus* IL-1β group.

Exogenous recombinant human IGF-1 markedly increased the phosphorylated IRS1 level in the chondrocytes after 30 min, while APS-3c did not (Figure 7 A). However, the pre-treated NVP-AEW541 significantly suppressed the phosphorylation of IRS-1 induced by IGF1 (Figure 7 A). Although GAG content and USSs mRNA expression was stimulated in chondrocytes treated with 10 µg/ml APS-3c, NVP-AEW541 significantly reduced the GAG synthesis to 66.1%, and *GALE*, *GALT*, *UGDH*, *UXS1* mRNA expression to 50.9%, 55.1%, 57.3% and 46.4% of the APS-3c group (Figure 7 B and C, *P*<0.05).

## Discussion

### APS-3c stimulated GAG synthesis in chondrocytes due to the up-regulated USSs gene expression

As GAG synthesis depends on the biosynthesis of UDP-sugar by USSs, modulating USSs gene expression or enzyme activity modulates GAG synthesis in chondrocytes. Over-expression of *UGDH* gene with retrovirus led to a remarkable increase of GAG synthesis and hyaluronic acid release in articular surface cells from chicken embryonic joints [38]. UXS1 activity is also essential for the production and organization of cartilage extracellular matrix [17]. Mutation of the *UXS1* gene resulted in a dramatic reduction and defective localization of proteoglycans in zebrafish, which consequently disturbed cartilage and bone morphogenesis [17]. In the present work, both *in vivo* and *in vitro* studies showed that ASP-3c obviously promoted GAG synthesis in the chondrocyte (Figure 1 and 3), which was mediated by the stimulated USSs gene expression (Figure 2 and 3). These findings are consistent with our previous work that APS-3c promoted expression of GTs genes and consequently enhanced GAG synthesis in rat primary chondrocytes [15]. However, how APS-3c stimulated gene expression of these USSs was still unknown.

### The up-regulation of USSs gene expression by ASP-3c was mediated by the enhanced gene expression of *IGF-1* and *IGF1R*

IGF-1 has long been proved essential in chondrogenesis, chondrocyte-phenotype maintenance and cartilage repair [39], [40]. IGF1 inhibited IL-1β-induced activation of NF-kB and apoptosis of chondrocytes partly through inhibiting phosphorylation, degradation and kinase activity of IkBα, which consequently inhibited NF-kB-mediated expression of some inflammation- and apoptosis-related genes [33], [41]. Meanwhile, IGF-1 also helped to retain the typical features in chondrocytes and enhances synthesis and release of PGs and collagen II mainly through activation of PI3K/Akt signaling pathway [29], [41], [42]. In the present study, exogenous IGF-1 promoted GAG synthesis and secretion in chondrocytes (Figure S2), increased mRNA level of USSs and abolished the inhibition of IL-1β on USSs mRNA expression (Figure 6), which agreed with the report by Maneix *et al* that IGF-1 up-stimulated *UGDH* gene expression and consequently promoted GAG synthesis in chondrocytes [43]. Interestingly, we also found that APS-3c stimulated both *IGF1* and *IGF1R* gene expression *in vivo* and *in vitro* (Figure 4 and 5), which indicated that the up-regulation of USSs gene expression by APS-3c was possibly mediated by IGF1/IGF1R pathway.

IRSs are signaling adaptor proteins which act as intermediates of the activated cell surface receptors, mostly the insulin receptor and IGF1R [44]. Binding of IGF1 to IGF1R leads to phosphorylation of IRS-1, and consequently the activation of the downstream signal cascades [26]. Here, we found that it was IGF1, not APS-3c, that induced an obvious increase of phosphorylated IRS-1 protein level within 30 min (Figure 7A), which indicated that APS-3c functioned mainly though up-regulating *IGF-1* and *IGF1R* gene expression but not through directly activating the IGF1R signaling pathway. Moreover, IGF1R inhibitor NVP-AEW541 markedly attenuated the USSs gene expression stimulated by APS-3c (Figure 7C). Altogether, these data indicated that promoted USSs gene expression and consequent GAG synthesis induced by APS-3c was due to the stimulated gene expression of *IGF-1* and *IGF1R* in the chondrocytes. This was further supported by our findings that APS-3c attenuated the IL-1β-induced degenerative ultrastructure of monolayer-cultured chondrocytes (Figure S3), as IGF-1 alone or co-treated with platelet-derived growth factor could attenuate the degenerative features and apoptosis induced by IL-1β in chondrocytes of monolayer culture [33]. Moreover, as APS-3c also helped to restore cartilage matrix, retain the integrality of cartilage surface and decrease the Mankin score of rat OA cartilage in our present and previous work [15], we guess that the cartilage-repair activities of ASP-3c were possibly mediated by the stimulation of *IGF1* and *IGF1R* gene expression.

### Take APS-3c as a novel SYSADOA?

APS-3c is a natural glycan with about 1.4×10^4^ Da molecular weight and containing 61.0% of sugars and 35.7% of uronic acids [14]. Sugar component of APS-3c was determined to be glucose, galactose, arabinose, rhamnose, mannose and xylose in a molar ratio of 6.3∶4.7∶6.7∶6.5∶1.6∶1.0 [14]. It’s reported that APS was a (1→4)-α-D-glucan with side chains at the glucosyl residues of the main chain [45], [46], indicating a similarity of the composition and molecular structure of APS-3c with chondroitin sulfate (CS), which is a natural sulfated GAG composed of the repeated disaccharide units of D-GlcA and D-GalNAc [47]. CS is a widely used SYSADOA, which partly modify, stabilize and postpone the pathology of OA [48], [49], [50], through promoting cartilage matrix synthesis and presenting anti-catabolic, anti-inflammatory, anti-apoptotic and anti-oxidant effects [47], [51]. In the present work, we found that APS-3c promoted GAG synthesis of articular chondrocytes and presented anti-inflammation and anti-degeneration effects against IL-1β through stimulating IGF1/IGF1R gene expression. Additionally, we also found that intra-gastric administration of APS-3c could increase the GAG content of OA rat cartilage and attenuate the inflammation of the OA synovium, but not affect the body weight of the rats (unpublished data). Moreover, APS-3c affected neither the cell viability of the primary chondrocytes (Figure S4), nor the integrality of normal rat cartilage [15]. Altogether, these data suggest that APS-3c might potentially be a novel SYSADOA.

## Conclusion

In conclusion, APS-3c promoted GAG synthesis and cartilage repair through stimulating USSs gene expression, which was mediated by the enhanced IGF1/IGF1R gene expression but not the direct activation of the IGF1R signaling pathway. Our findings presented a better understanding of the pharmacological effects of APS-3c on OA and suggested that APS-3c might potentially be a novel SYSADOA.

## Supporting Information

Figure S1 ***Angelica sinensis*** **polysaccharides (APS-3c) stimulated glycosaminoglycan (GAG) synthesis and secretion of human primary chondrocyte.** A, Chondrocytes-associated GAG was stained with Alcian blue dye (original magnification of 200); B, The optical density analysis of Alcian blue positive spots was performed using NIS-Elements software (Nikon, Tokyo, Japan). Values are presented as mean ± SEM from at least three independent experiments. *P<0.05, **P<0.01 *versus* control group; ^#^ *P*<0.05, ^##^ *P*<0.01*versus* IL-1β group.
(TIF)

Figure S2 **Insulin like growth factor 1 (IGF1) stimulated glycosaminoglycan (GAG) synthesis of human primary chondrocytes.** Chondrocytes were treated with IGF-1 (2, 10 and 50 ng/ml) or IL-1β (10 ng/ml) alone for 48 h, respectively. Meanwhile, chondrocytes were pre-treated with IL-1β (10 ng/ml) for 30 min and then co-treated with IL-1β and IGF-1 (2, 10 and 50 ng/ml) for another 48 h. Then, 1,9-dimethylmethylene Blue was applied to detect the GAG of chondrocyte cultures. Values are presented as mean ± SEM from at least two independent experiments. *P<0.05, **P<0.01 *versus* control group;^ #^ *P*<0.05, ^##^ *P*<0.01*versus* IL-1β group.
(TIF)

Figure S3 **Effects of** ***Angelica sinensis*** **polysaccharides (APS-3c) on the ultrastructure of human primary chondrocytes.** A, Control group; B–D, Chondrocytes treated with 2, 10 and 50 µg/ml APS-3c for 48 h; E, Chondrocytes induced by 10 ng/ml IL-1β for 48 h;F–H, Chondrocytes pre-treated with 10 ng/ml IL-1β for 30 min and then co-treated with IL-1β and 2, 10 and 50 µg/ml APS-3c for 48 h. All photographs were taken at an original magnification of 15,000. N, nucleus;M, mitochondrion; rER, rough surfaced endoplasmic reticulum; G, Golgi apparatus; V, vacuole; AV, autophagic vacuole; SV, secretory vesicle.
(TIF)

Figure S4 **Effects of** ***Angelica sinensis*** **polysaccharides (APS-3c) on cell viability of human primary chondrocytes.** Chondrocytes were treated with 2, 10 and 50 µg/ml APS-3c for 48 h or pre-treated with IL-1β (10 ng/ml) for 30 min, and then co-treated with IL-1β and APS-3c (2, 10 and 50 µg/ml) for 48 h. Values are presented as mean ± SEM from at least three independent experiments. *P<0.05*versus* control group.
(TIF)
