# Supplementary figures and images for: Angelica Sinensis Polysaccharides Stimulated UDP-Sugar Synthase Genes through Promoting Gene Expression of IGF-1 and IGF1R in Chondrocytes: Promoting Anti-Osteoarthritic Activity
Source: PLoS One. 2014 Sep 9;9(9):e107024. doi: 10.1371/journal.pone.0107024 (PMC4159308; doi:10.1371/journal.pone.0107024)

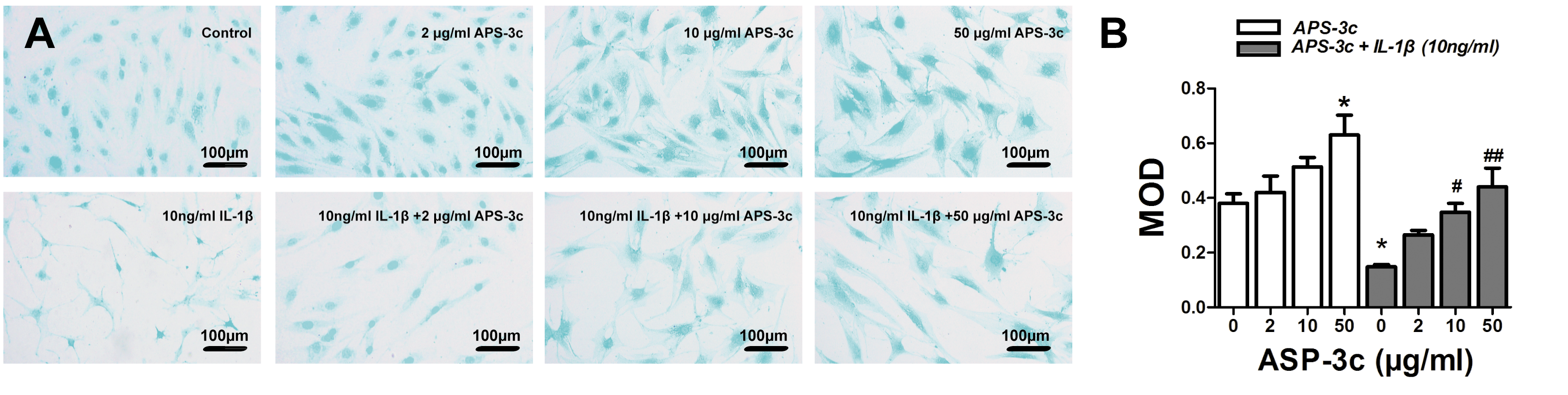

Supplement: Figure S1 — Angelica sinensis polysaccharides (APS-3c) stimulated glycosaminoglycan (GAG) synthesis and secretion of human primary chondrocyte. A, Chondrocytes-associated GAG was stained with Alcian blue dye (original magnification of 200); B, The optical density analysis of Alcian blue positive spots was performed using NIS-Elements software (Nikon, Tokyo, Japan). Values are presented as mean ± SEM from at least three independent experiments. *P<0.05, **P<0.01 versus control group; # P<0.05, ## P<0.01versus IL-1β group. (TIF) [file pone.0107024.s001.tif]

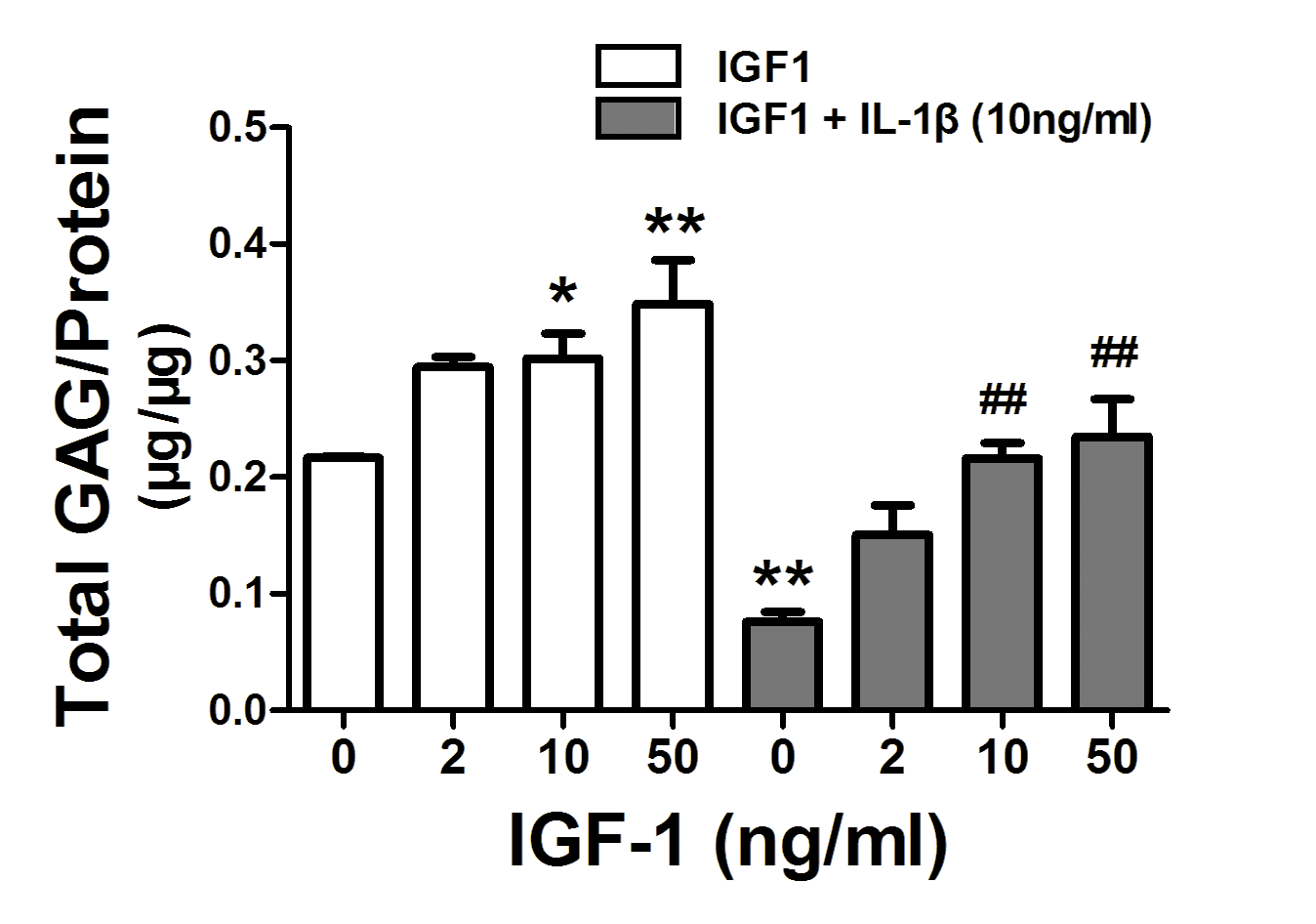

Supplement: Figure S2 — Insulin like growth factor 1 (IGF1) stimulated glycosaminoglycan (GAG) synthesis of human primary chondrocytes. Chondrocytes were treated with IGF-1 (2, 10 and 50 ng/ml) or IL-1β (10 ng/ml) alone for 48 h, respectively. Meanwhile, chondrocytes were pre-treated with IL-1β (10 ng/ml) for 30 min and then co-treated with IL-1β and IGF-1 (2, 10 and 50 ng/ml) for another 48 h. Then, 1,9-dimethylmethylene Blue was applied to detect the GAG of chondrocyte cultures. Values are presented as mean ± SEM from at least two independent experiments. *P<0.05, **P<0.01 versus control group; # P<0.05, ## P<0.01versus IL-1β group. (TIF) [file pone.0107024.s002.tif]

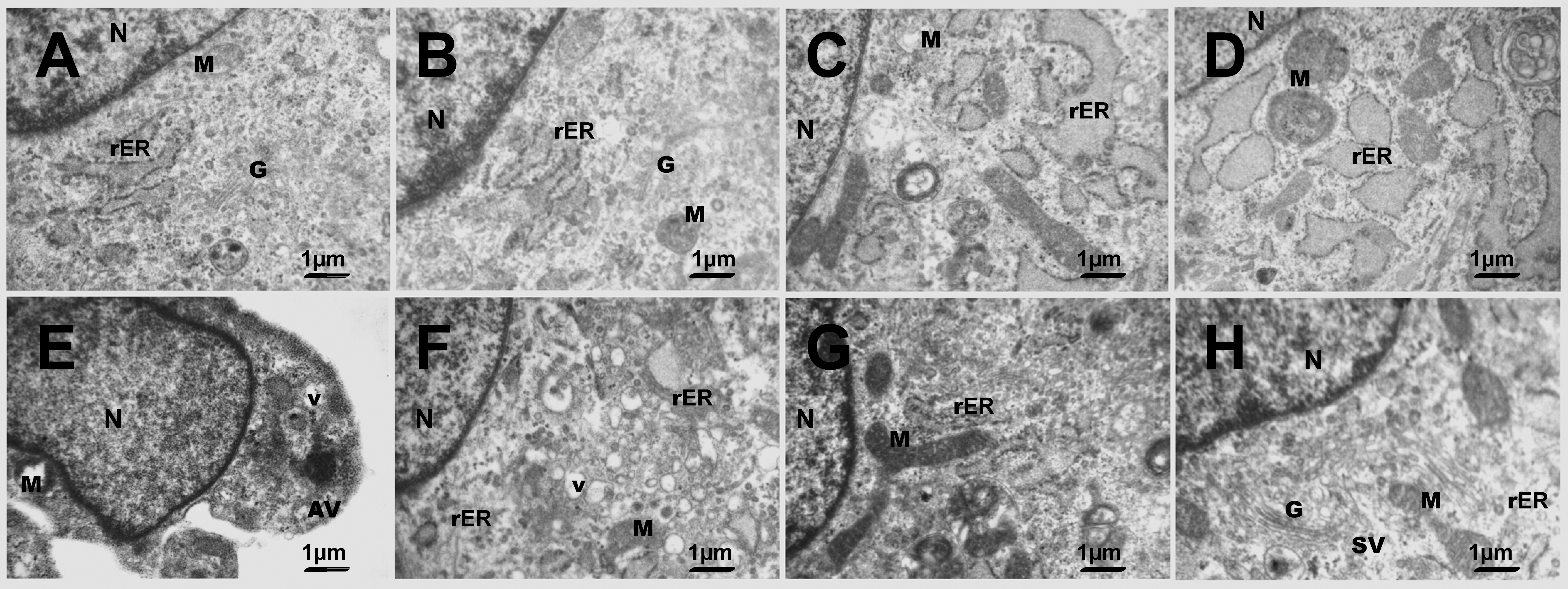

Supplement: Figure S3 — Effects of Angelica sinensis polysaccharides (APS-3c) on the ultrastructure of human primary chondrocytes. A, Control group; B–D, Chondrocytes treated with 2, 10 and 50 µg/ml APS-3c for 48 h; E, Chondrocytes induced by 10 ng/ml IL-1β for 48 h;F–H, Chondrocytes pre-treated with 10 ng/ml IL-1β for 30 min and then co-treated with IL-1β and 2, 10 and 50 µg/ml APS-3c for 48 h. All photographs were taken at an original magnification of 15,000. N, nucleus;M, mitochondrion; rER, rough surfaced endoplasmic reticulum; G, Golgi apparatus; V, vacuole; AV, autophagic vacuole; SV, secretory vesicle. (TIF) [file pone.0107024.s003.tif]

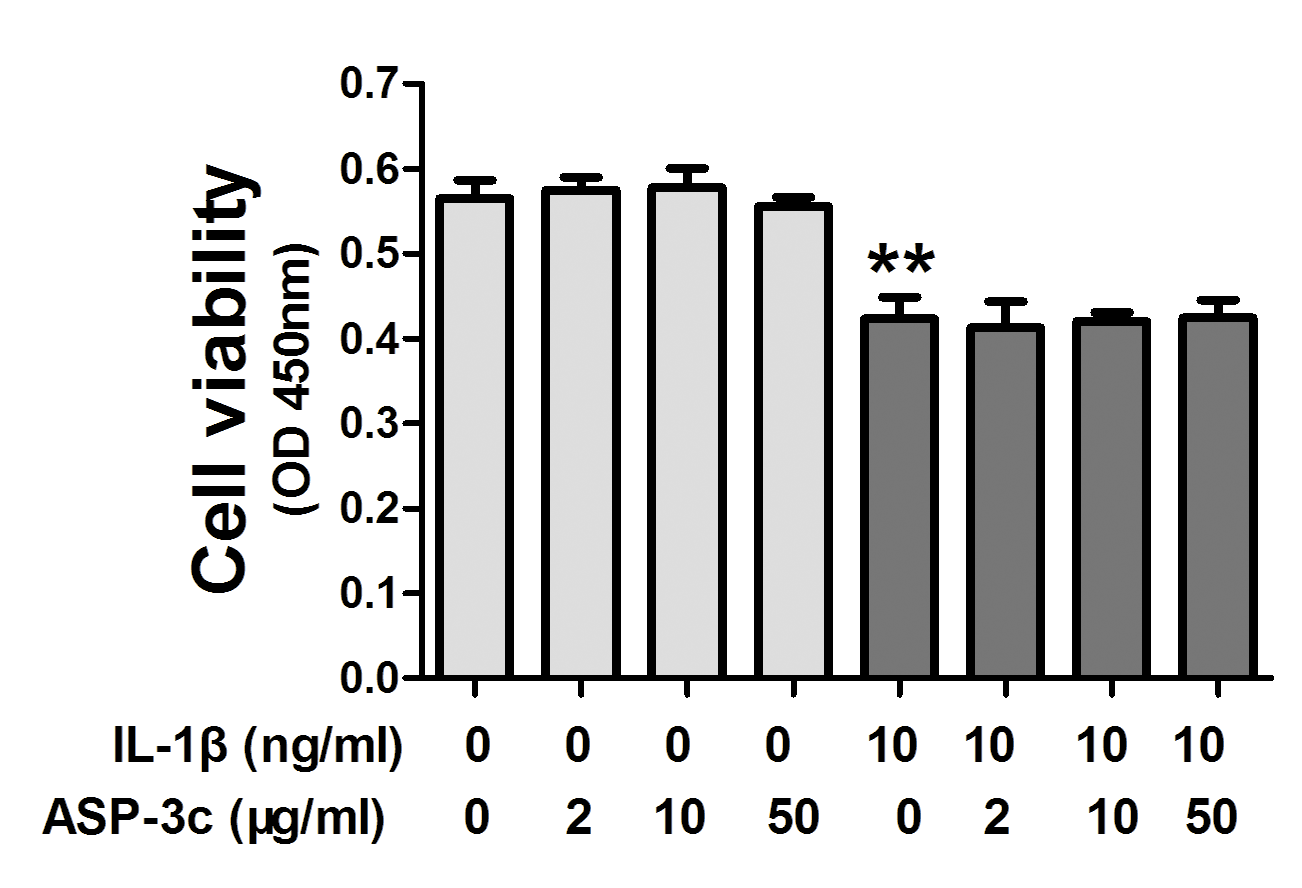

Supplement: Figure S4 — Effects of Angelica sinensis polysaccharides (APS-3c) on cell viability of human primary chondrocytes. Chondrocytes were treated with 2, 10 and 50 µg/ml APS-3c for 48 h or pre-treated with IL-1β (10 ng/ml) for 30 min, and then co-treated with IL-1β and APS-3c (2, 10 and 50 µg/ml) for 48 h. Values are presented as mean ± SEM from at least three independent experiments. *P<0.05versus control group. (TIF) [file pone.0107024.s004.tif]
